# Trajectories of post-stroke fatigue and its influencing factors in young and middle-aged patients with intracerebral hemorrhage

**DOI:** 10.3389/fneur.2026.1921237

**Published:** 2026-09-24

**Authors:** Hui Qiu, Lixia Du, Xiaopeng Shi, Yanwei Cheng, Lijun Xu, Shuting Zang

**Affiliations:** 1 Department of Emergency, Henan Provincial People’s Hospital, People's Hospital of Zhengzhou University, Zhengzhou, Henan, China; 2 Department of Emergency, Henan Provincial Key Medicine Laboratory of Nursing, Henan Provincial People’s Hospital, Zhengzhou, Henan, China; 3 Zhengzhou University People’s Hospital, Henan University People’s Hospital, Zhengzhou, Henan, China

**Keywords:** fatigue trajectory, intracerebral hemorrhage, latent class growth modeling, nursing care, post-stroke fatigue, predictors, young and middle-aged adults

## Abstract

**Objective:**

To explore the developmental trajectories of post-stroke fatigue and its influencing factors in young and middle-aged patients with intracerebral hemorrhage, and to provide theoretical basis for precise clinical nursing interventions.

**Methods:**

A convenience sampling method was used to enroll 218 young and middle-aged patients with intracerebral hemorrhage who were admitted to the Emergency Intensive Care Unit (EICU) between July 2022 and July 2024. Assessments were conducted at four time points: within 24 h after neurological stabilization during the acute phase (T1), and at 1 month (T2), 6 months (T3), and 12 months (T4) post-discharge. The Fatigue Severity Scale (FSS), Connor-Davidson Resilience Scale (CD-RISC), Medical Outcomes Study Social Support Survey (MOS-SSS), and Fear of Progression Questionnaire-Short Form (FoP-Q-SF) were administered. Latent class growth modeling (LCGM) was used to identify the developmental trajectories of fatigue based on the 218 enrolled participants with Full Information Maximum Likelihood handling of missing data, and multinomial logistic regression was applied to analyze the influencing factors of trajectory group membership among the 180 participants who completed all four assessments.

**Results:**

(1) Three heterogeneous developmental trajectories of post-stroke fatigue were identified in young and middle-aged patients with intracerebral hemorrhage: low-level stable fatigue group (30.6%), moderate-level slowly resolving group (43.3%), and high-level persistent fatigue group (26.1%). (2) Multinomial logistic regression analysis showed that, using the low-level stable fatigue group as the reference, increasing age (OR = 1.085, *p* = 0.007; OR = 1.132, *p* = 0.001), having ≥2 chronic comorbidities (OR = 2.186, *p* = 0.014; OR = 3.418, p = 0.001), and higher NIHSS score (OR = 1.118, *p* = 0.004; OR = 1.194, *p* < 0.001) were common independent risk factors for entering both the moderate-level slowly resolving group and the high-level persistent fatigue group; psychological resilience (OR = 0.951, *p* = 0.003; OR = 0.924, *p* < 0.001) and social support (OR = 0.936, *p* = 0.004; OR = 0.908, *p* = 0.001) were common protective factors. Elevated fear of disease progression (OR = 1.112, *p* = 0.001) was an independent risk factor for entering the high-level persistent fatigue group, while having a college degree or above (OR = 0.395, *p* = 0.022) was an independent protective factor against entering the high-level persistent fatigue group.

**Conclusion:**

There are three heterogeneous developmental trajectories of post-stroke fatigue in young and middle-aged patients with intracerebral hemorrhage. Although fatigue levels in most patients showed a resolving trend over time, approximately one-quarter of patients still exhibited persistent high-level fatigue. Healthcare providers should implement stratified and dynamic precision interventions based on different trajectory characteristics, with particular attention to patients who are older, have multiple comorbidities, severe neurological deficits, and high levels of fear of disease progression, while also considering patients’ psychological resilience and social support during rehabilitation planning to promote effective fatigue relief.

## 1 Introduction

Stroke is a neurological disorder that poses a serious threat to the health of young and middle-aged populations, with an increasing trend of younger onset in recent years. Unlike elderly patients, young and middle-aged individuals, as the core labor force of both society and family, have extremely high expectations for neurological recovery and social reintegration (1, 2). However, among the complex symptom clusters following stroke, including limb dysfunction, cognitive decline, and negative emotions (3), post-stroke fatigue (PSF) has garnered increasing attention due to its high incidence and persistence.

PSF is defined as “a feeling of exhaustion, weariness or lack of energy that can be overwhelming, and which can involve physical, emotional, cognitive and perceptual contributors, which is not relieved by rest and affects a person’s daily life” (4). It not only severely undermines patients’ adherence to rehabilitation training and reduces their quality of daily life, but also serves as an independent risk factor for stroke recurrence and mortality, significantly increasing the burden of family caregiving (5). The overall prevalence of PSF in China ranges from 23 to 74% (6), and among young and middle-aged populations in particular, the incidence rate is as high as 62.3%, directly leading to decreased social participation and difficulties in returning to work (7).

Currently, domestic and international research on PSF has largely focused on elderly populations with ischemic stroke, with the majority employing cross-sectional designs (8, 9) that can only describe fatigue levels at a single time point and fail to reveal the dynamic patterns of individual patients’ fatigue changes throughout the rehabilitation period. It is noteworthy that there are significant differences in pathophysiological mechanisms between intracerebral hemorrhage and cerebral infarction. Intracerebral hemorrhage is more commonly accompanied by headache, nausea, vomiting, and depressed level of consciousness at onset due to increased intracranial pressure and brain displacement from the mass effect of the hematoma (10). Scopelliti et al. (11) reported that fatigue after spontaneous intracerebral hemorrhage is frequent and long-lasting, with 51% of survivors reporting fatigue at 1-year follow-up. Moreover, Juárez-Belaúnde et al. (5) observed differing patterns of fatigue progression between ischemic and hemorrhagic strokes: fatigue tends to ameliorate over time in hemorrhagic stroke cases, paralleling functional recovery, while it remains stable over time in ischemic stroke cases. However, longitudinal studies on fatigue specifically targeting young and middle-aged patients with intracerebral hemorrhage are still lacking.

Recent longitudinal studies have examined post-stroke depressive symptom changes and symptom dynamics using cross-lagged panel network analysis, revealing that depressive symptoms, anxiety, fatigue, sleep disturbance, and stigma may exhibit complex trajectory patterns over time (9). These findings provide important methodological and clinical context for the present trajectory-based fatigue study. Understanding how fatigue trajectories differ from, overlap with, or contribute to other post-stroke psychological symptom patterns may inform more comprehensive rehabilitation strategies.

Based on the Chronic Illness Trajectory Framework (12), this study adopts a longitudinal design to investigate the developmental trajectories of post-stroke fatigue and its heterogeneity in young and middle-aged patients with intracerebral hemorrhage, and to further analyze its key influencing factors, with the aim of providing a theoretical basis for clinical nursing staff to implement precise and dynamic fatigue intervention strategies.

## 2 Methods

### 2.1 Study design and participants

This was a prospective longitudinal observational study. Using a convenience sampling method, young and middle-aged patients with hemorrhagic stroke hospitalized in the Department of Emergency Intensive Care Unit (EICU) of our hospital from July 2022 to July 2024 were enrolled as study participants.

*Inclusion criteria*: (1) first-ever intracerebral hemorrhage confirmed by cranial CT or MRI; (2) age ≥18 years and ≤59 years, based on the World Health Organization’s definition of young and middle-aged adults and consistent with previous studies on stroke in this population; (3) stable vital signs, with basic language comprehension and communication skills to complete the questionnaire assessments.

*Exclusion criteria*: (1) complicated with malignant tumors or severe cardiac, hepatic, or renal failure; (2) complicated with immune system diseases, sleep apnea syndrome, or other conditions clearly leading to secondary fatigue; (3) history of psychiatric disorders or severe cognitive impairment (Mini-Mental State Examination score <24) that precluded independent completion of questionnaires; (4) complicated with drug or alcohol dependence; (5) concurrent participation in other clinical studies.

All enrolled patients or their legal representatives signed written informed consent. This study was approved by the Hospital Medical Ethics Committee (approval number: H20230842) and complied with the requirements of the Declaration of Helsinki.

### 2.2 Sample size estimation

Latent class growth modeling (LCGM) was used for trajectory analysis. Referring to the rule of thumb proposed by Nylund et al. (13), the sample size should be at least 10 to 20 times the number of independent variables, with a 20% attrition rate considered. This study planned to include 12 core predictive variables, and based on a 1:15 ratio, the required sample size was 180 cases. A total of 286 patients were initially screened, of whom 52 were excluded and 16 declined participation, resulting in 218 patients enrolled at T1 baseline. During the four follow-up visits, 38 patients were lost to follow-up, yielding an attrition rate of 17.4% (38/218). Ultimately, 180 patients completed all four follow-ups and were included in the final analysis, meeting the model fitting requirements. A comparison of baseline characteristics between retained participants (*n* = 180) and those lost to follow-up (*n* = 38) showed no statistically significant differences in age, sex, NIHSS score, or FSS score at T1 (all *p* > 0.05), suggesting that attrition bias was unlikely to substantially affect the results. The patient enrollment process is shown in Figure 1.

**Figure 1:**
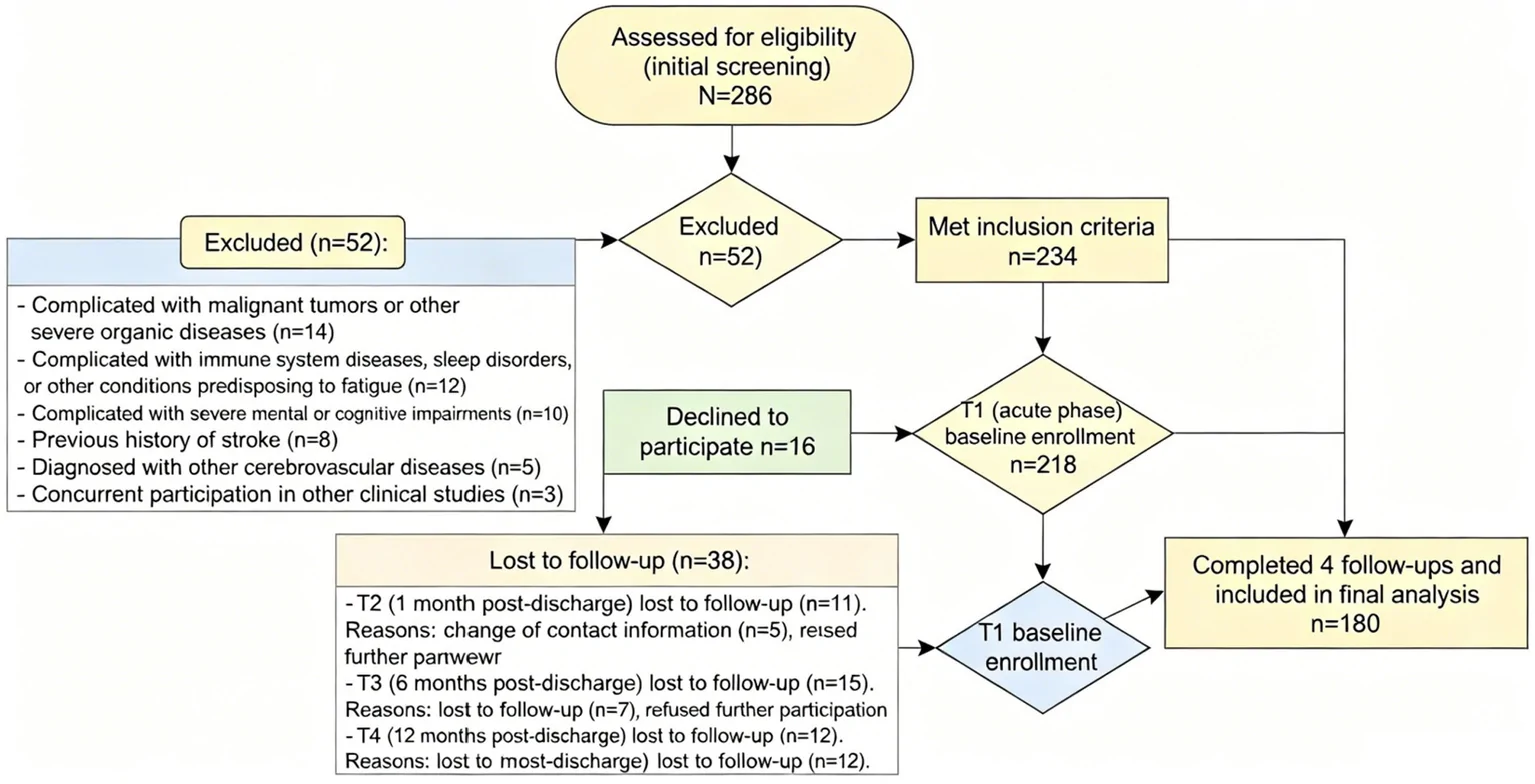
Flowchart of study participant screening.

### 2.3 Data collection methods and quality control

Four time points were set for follow-up assessments: T1 (acute phase, within 24 h after neurological stabilization), T2 (1 month post-discharge), T3 (6 months post-discharge), and T4 (12 months post-discharge). At T1, two uniformly trained researchers used standardized instructions to explain the study purpose and questionnaire completion requirements to patients, administered paper questionnaires, and collected them on-site after patients completed them independently. For patients unable to write due to limb dysfunction, the researchers read the questions aloud item by item and recorded the patients’ choices. At T2–T4, questionnaire data were collected through outpatient follow-up visits or telephone interviews. The FSS has been successfully administered via telephone in previous stroke studies, and a recent study demonstrated good agreement between face-to-face and telephone administration of the FSS in elderly populations (14), supporting the validity of telephone-based data collection in this study. For quality control, all questionnaires were immediately checked upon collection, and any missing items were supplemented on-site by the patients. Data were independently double-entered by two researchers using EpiData 3.1 software and cross-validated to ensure data accuracy.

### 2.4 Research instruments

#### 2.4.1 General information and clinical data questionnaire

A self-designed questionnaire was used to collect: (1) demographic characteristics, including age, sex, educational level, marital status, average monthly family income, place of residence, and medical payment method; (2) lifestyle factors, including smoking history and alcohol consumption history; (3) clinical characteristics, including the number of chronic comorbidities (diabetes, hypertension, etc.), body mass index (BMI), and family history of stroke.

#### 2.4.2 Assessment of neurological function and disability

(1) The National Institutes of Health Stroke Scale (NIHSS) was used to assess the severity of neurological deficits. The scale consists of 11 items, with a total score ranging from 0 to 42; higher scores indicate more severe neurological deficits. In this study, the NIHSS was completed by the attending neurologist upon patient admission.
(2) The modified Rankin Scale (mRS) was used to assess the degree of functional disability. The scale has 7 grades (0–6), with 0 indicating no symptoms and 6 indicating death; higher scores indicate greater disability. Previous studies have shown good inter-rater reliability for this scale, with a weighted Kappa value of 0.91.

#### 2.4.3 Fatigue severity scale (FSS)

The FSS was used to assess the severity of post-stroke fatigue. The scale consists of 9 items, each scored on a 7-point Likert scale (1–7). The total score is the mean of the 9 item scores, with a score >4 indicating the presence of post-stroke fatigue. Higher scores indicate more severe fatigue. The Chinese version of the FSS was translated and validated by Wu and Wang (15) in a sample of 153 stroke patients, demonstrating good reliability (Cronbach’s *α* = 0.929) and validity. In this study, the Cronbach’s α coefficient of this scale was 0.93.

#### 2.4.4 Connor-Davidson resilience scale (CD-RISC)

The CD-RISC was used to assess patients’ psychological resilience. The scale comprises three dimensions (tenacity, strength, and optimism) with a total of 25 items, each scored on a 5-point Likert scale (0–4). The total score ranges from 0 to 100, with higher scores indicating better psychological resilience. The Chinese version of the CD-RISC has demonstrated good psychometric properties in Chinese populations, with a reported Cronbach’s *α* of 0.91, and has been validated in stroke survivors (16). In this study, the Cronbach’s α coefficient of this scale was 0.91.

#### 2.4.5 Medical outcomes study social support survey (MOS-SSS)

The MOS-SSS was used to assess patients’ social support levels. The scale contains 19 items covering dimensions such as tangible support, emotional support, informational support, and positive social interaction, with each item scored on a 5-point Likert scale. Higher scores indicate higher levels of perceived social support. The Chinese version of the MOS-SSS (MOS-SSS-C) has been psychometrically tested and validated, with confirmatory factor analysis affirming its four-factor structure and Cronbach’s alphas for subscales ranging from 0.93 to 0.96 (17). In this study, the Cronbach’s *α* coefficient of this scale was 0.92.

#### 2.4.6 Fear of progression questionnaire-short form (FoP-Q-SF)

The FoP-Q-SF was used to assess patients’ fear of disease progression. The scale comprises two dimensions (physical health and social/family) with a total of 12 items, each scored on a 5-point Likert scale (1–5). The total score ranges from 12 to 60, with higher scores indicating higher levels of fear. The simplified Mandarin version of the FoP-Q-SF has been validated for use in Chinese populations, demonstrating high internal and test–retest reliability (18). In this study, the Cronbach’s *α* coefficient of this scale was 0.88.

It should be noted that psychological resilience, social support, and fear of disease progression were measured only at baseline (T1). Thus, causal interpretations regarding these factors and fatigue trajectory membership should be made with caution, as temporal precedence cannot be firmly established.

### 2.5 Statistical analysis

Data analysis was performed using SPSS 25.0 and Mplus 8.7 software. Continuous variables following a normal distribution were expressed as mean ± standard deviation (mean ± SD), while those not following a normal distribution were expressed as median (interquartile range) [M (P₂₅, P₇₅)]. Categorical variables were expressed as frequencies (percentages) [*n* (%)].

(1) Latent class growth modeling (LCGM): LCGM was constructed using Mplus 8.7 based on FSS scores at the four time points to identify different developmental trajectory subtypes of post-stroke fatigue. In model specification, both linear and quadratic trajectories were tested. Intercepts and slopes were specified as random effects with freely estimated variances, allowing individual variability around the class-specific mean trajectories. The optimal model was selected based on a combination of statistical fit indices and clinical interpretability. Model fit evaluation indices included Akaike Information Criterion (AIC), Bayesian Information Criterion (BIC), and sample-size adjusted BIC (aBIC), with smaller values indicating better model fit. Entropy was used to assess classification accuracy, with values >0.8 indicating classification precision >90%. The Lo–Mendell–Rubin (LMR) test and the Bootstrap Likelihood Ratio Test (BLRT) were used to compare the fit differences between k-class and (k-1)-class models, with *p* < 0.05 indicating that the k-class model was significantly better than the (k-1)-class model. The optimal number of trajectory classes was determined by comprehensively considering the statistical indices and clinical interpretability.
(2) Analysis of influencing factors: The trajectory class groups identified by LCGM were used as the dependent variable (categorical variable). Univariate analysis was first performed: continuous variables following a normal distribution were analyzed using one-way analysis of variance (ANOVA), while those not following a normal distribution were analyzed using the Kruskal-Wallis H test; categorical variables were analyzed using the chi-square (*χ*^2^) test or Fisher’s exact test. Variables with *p* < 0.10 in the univariate analysis were included in a multinomial logistic regression model using the forward stepwise method to identify independent factors influencing different fatigue trajectory subtypes. Results were presented as odds ratios (OR) with 95% confidence intervals (CI). The Hosmer-Lemeshow test, which has been extended for multinomial logistic regression models, was used to assess model goodness-of-fit.
(3) Missing data handling: For the LCGM analysis, all 218 participants enrolled at baseline were included, and the Full Information Maximum Likelihood (FIML) method was used for parameter estimation, which utilizes all available data to reduce bias due to missing data. For the multinomial logistic regression analysis, only the 180 participants who completed all four assessments were included, as trajectory class membership required complete data across all time points. For item missingness occurring during the four follow-ups, the Full Information Maximum Likelihood (FIML) method was used for parameter estimation, which utilizes all available data to reduce bias due to missing data.

The significance level was set at *α* = 0.05 (two-sided), and *p* < 0.05 was considered statistically significant.

## 3 Results

### 3.1 General characteristics of the study participants

A total of 180 young and middle-aged patients with intracerebral hemorrhage were enrolled in this study, including 112 males (62.2%) and 68 females (37.8%). The age ranged from 22 to 59 years, with a mean of (46.3 ± 9.1) years. Regarding educational level, 62 cases (34.4%) had junior high school education or below, 58 cases (32.2%) had high school or secondary technical school education, and 60 cases (33.3%) had college degree or above. Regarding marital status, 154 cases (85.6%) were married, and 26 cases (14.4%) were unmarried/divorced/widowed. Regarding chronic comorbidities, 142 cases (78.9%) had hypertension, 51 cases (28.3%) had diabetes, and 67 cases (37.2%) had two or more chronic comorbidities. A total of 78 cases (43.3%) had a history of smoking, and 55 cases (30.6%) had a history of alcohol consumption. The median NIHSS score at admission was 9.0 (P₂₅ = 5.0, P₇₅ = 15.0). Regarding GCS scores, 138 cases (76.7%) scored 13–15, 32 cases (17.8%) scored 9–12, and 10 cases (5.6%) scored 3–8. Regarding hemorrhage location, 96 cases (53.3%) had basal ganglia hemorrhage, 42 cases (23.3%) had lobar hemorrhage, 18 cases (10.0%) had brainstem hemorrhage, 14 cases (7.8%) had cerebellar hemorrhage, and 10 cases (5.6%) had hemorrhage at other sites. The mean length of hospital stay was (18.5 ± 8.3) days (Table 1).

**Table 1:** General characteristics of young and middle-aged patients with intracerebral hemorrhage (*N* = 180).

Variable	Category	*n* (%)/(mean ± SD)
Demographic characteristics		
Age (years)	22–59	46.3 ± 9.1
Age group	<40 years	42 (23.3)
	40–49 years	68 (37.8)
	≥50 years	70 (38.9)
Sex	Male	112 (62.2)
	Female	68 (37.8)
Educational level	Junior high school or below	62 (34.4)
	High school/technical secondary school	58 (32.2)
	College degree or above	60 (33.3)
Marital status	Married	154 (85.6)
	Unmarried/divorced/widowed	26 (14.4)
Average monthly family income	<3,000 RMB	48 (26.7)
	3,000–5,000 RMB	72 (40.0)
	>5,000 RMB	60 (33.3)
Place of residence	Urban	98 (54.4)
	Rural	82 (45.6)
Medical payment method	Urban employee medical insurance	62 (34.4)
	Urban–rural resident medical insurance	96 (53.3)
	Self-pay	22 (12.2)
Lifestyle factors		
Smoking history	Yes	78 (43.3)
	No	102 (56.7)
Alcohol consumption history	Yes	55 (30.6)
	No	125 (69.4)
Clinical characteristics		
Hypertension history	Yes	142 (78.9)
	No	38 (21.1)
Diabetes history	Yes	51 (28.3)
	No	129 (71.7)
Number of chronic comorbidities	0	48 (26.7)
	1	65 (36.1)
	≥2	67 (37.2)
BMI (kg/m^2^)	—	24.6 ± 3.2
BMI group	<18.5 (underweight)	8 (4.4)
	18.5–23.9 (normal)	76 (42.2)
	24.0–27.9 (overweight)	68 (37.8)
	≥28.0 (obese)	28 (15.6)
Family history of stroke	Yes	22 (12.2)
	No	158 (87.8)
Length of hospital stay (days)	—	18.5 ± 8.3
Disease severity		
NIHSS score	—	9.0 (5.0, 15.0)
GCS grade	13–15 (mild)	138 (76.7)
	9–12 (moderate)	32 (17.8)
	3–8 (severe)	10 (5.6)
Hemorrhage location	Basal ganglia	96 (53.3)
	Lobar	42 (23.3)
	Brainstem	18 (10.0)
	Cerebellum	14 (7.8)
	Others	10 (5.6)

The FSS scores of the 180 patients at the four time points were: T1 (5.48 ± 1.42), T2 (4.99 ± 1.38), T3 (4.49 ± 1.47), and T4 (4.02 ± 1.56), showing an overall downward trend. Repeated-measures ANOVA showed that the differences were statistically significant (*F* = 54.72, *p* < 0.001).

### 3.2 Developmental trajectories of post-stroke fatigue in young and middle-aged patients with intracerebral hemorrhage

Latent class growth modeling (LCGM) was used to fit the trajectories of FSS scores at the four time points in 180 patients. Models with 1 to 4 classes were fitted separately, and the model fit information is shown in Table 2. The results showed that the AIC, BIC, and aBIC of the 3-class model decreased significantly compared with the 2-class model, with an Entropy value of 0.895 (>0.8). Both the LMR and BLRT tests were significant (*p* < 0.001), indicating that the 3-class model had the best classification accuracy. The LMR test for the 4-class model was not significant (*p* = 0.082), suggesting that adding a fourth class did not significantly improve model fit. Based on the comprehensive evaluation of fit indices and clinical interpretability, the optimal number of classes for the developmental trajectories of post-stroke fatigue in young and middle-aged patients with intracerebral hemorrhage was determined to be 3.

**Table 2:** Fit indices of LCGM models with different numbers of classes.

Number of classes	AIC	BIC	aBIC	Entropy	LMR (P)	BLRT (P)	Class proportions (%)
1 Class	2862.47	2888.30	2864.03	—	—	—	100
2 Classes	2696.21	2735.38	2699.59	0.848	<0.001	<0.001	47.8/52.2
3 Classes	2558.06	2610.57	2563.26	0.895	<0.001	<0.001	30.6/43.3/26.1
4 Classes	2544.32	2610.17	2551.34	0.879	0.082	0.068	26.7/31.1/25.0/17.2

Based on the FSS score levels and change trends of the three trajectories, they were, respectively, named the low-level stable fatigue group (Class 1), the moderate-level slowly resolving group (Class 2), and the high-level persistent fatigue group (Class 3). Specifically, Class 1 comprised 55 cases (30.6%). The FSS scores of this group remained at consistently low levels from T1 to T4 (T1: 3.85 ± 0.92, T2: 3.42 ± 0.85, T3: 3.08 ± 0.78, T4: 2.85 ± 0.72), with all time points below the cutoff value for fatigue (4 points) and with little overall fluctuation, indicating mild and stable fatigue. Class 2 comprised 78 cases (43.3%). The FSS scores of this group were at moderate levels at T1 (5.62 ± 0.88), gradually decreased with rehabilitation time, remained at relatively high levels at T2 (5.02 ± 1.02), approached the cutoff at T3 (4.38 ± 1.12), and dropped below the cutoff at T4 (3.75 ± 1.08), showing a slowly resolving trend. Class 3 comprised 47 cases (26.1%). The FSS scores of this group reached a high level of (7.15 ± 0.65) at T1. Although there was a slight decline over time (T4: 5.85 ± 1.04), all time points remained significantly above the cutoff (>4 points), indicating severe and persistent fatigue with slow recovery. The differences in FSS scores among the three groups at all four time points were statistically significant (*p* < 0.001). The FSS scores of the three groups at each time point are detailed in Table 3 and Figure 2.

**Table 3:** Comparison of FSS scores at each time point among different trajectory groups (mean ± SD).

Group	*n* (%)	T1	T2	T3	T4	*F*	*p*
Class 1 (Low-level stable)	55 (30.6)	3.85 ± 0.92	3.42 ± 0.85	3.08 ± 0.78	2.85 ± 0.72	14.26	<0.001
Class 2 (Moderate-level resolving)	78 (43.3)	5.62 ± 0.88	5.02 ± 1.02	4.38 ± 1.12	3.75 ± 1.08	46.83	<0.001
Class 3 (High-level persistent)	47 (26.1)	7.15 ± 0.65	6.78 ± 0.82	6.32 ± 0.95	5.85 ± 1.04	28.57	<0.001
Total	180	5.48 ± 1.42	4.99 ± 1.38	4.49 ± 1.47	4.02 ± 1.56	54.72	<0.001
*F*		178.56	156.34	128.67	98.45		
*p*		<0.001	<0.001	<0.001	<0.001		

**Figure 2:**
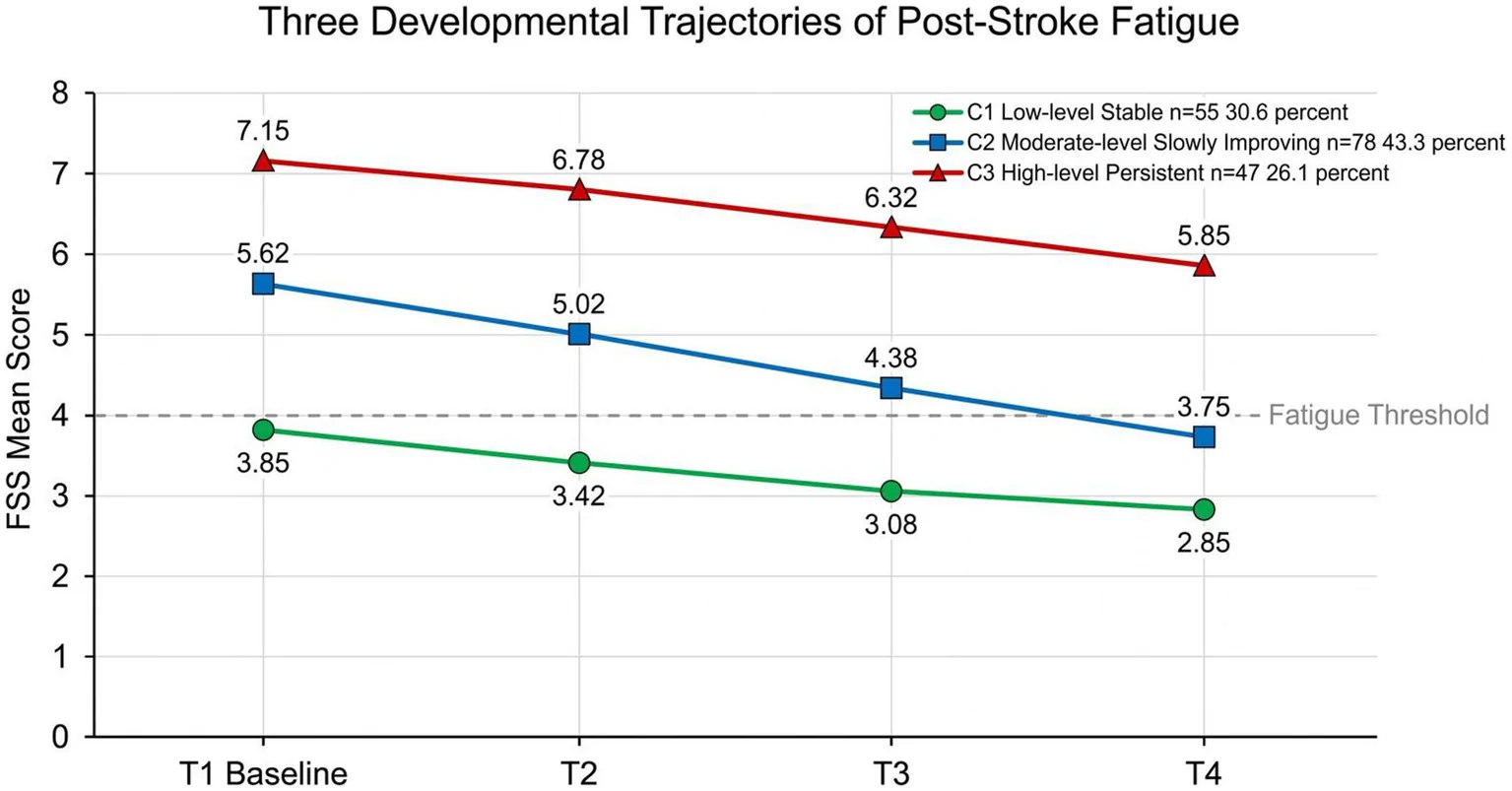
Three developmental trajectories of post-stroke fatigue in young and middle-aged patients with intracerebral hemorrhage.

### 3.3 Univariate analysis of factors influencing post-stroke fatigue developmental trajectories in young and middle-aged patients with intracerebral hemorrhage

Using trajectory group classification (Class 1, Class 2, Class 3) as the dependent variable and patients’ general and clinical characteristics as independent variables, univariate analysis was performed. The results showed that age, educational level, marital status, number of chronic comorbidities, hypertension history, NIHSS score, GCS grade, psychological resilience (CD-RISC) total score, social support (MOS-SSS) total score, and fear of disease progression (FoP-Q-SF) total score showed statistically significant differences among the three groups (*p* < 0.05). Sex, smoking history, alcohol consumption history, BMI, family history of stroke, and hemorrhage location showed no statistically significant differences among the three groups (*p* > 0.05). The results of the univariate analysis are shown in Table 4.

**Table 4:** Univariate analysis of different trajectory groups of post-stroke fatigue in young and middle-aged patients with intracerebral hemorrhage.

Variable	Class 1 (*n* = 55)	Class 2 (*n* = 78)	Class 3 (*n* = 47)	Statistic (*F*/*χ*^2^/*H*)	*p*
Age (years, mean ± SD)	41.2 ± 8.5	47.1 ± 8.2	50.3 ± 7.6	17.42^a^	<0.001
Sex (male/female)	34/21	49/29	29/18	0.062^b^	0.970
Educational level (%)				22.31^b^	<0.001
Junior high school or below	14 (25.5)	28 (35.9)	20 (42.6)		
High school/technical secondary school	18 (32.7)	26 (33.3)	14 (29.8)		
College degree or above	23 (41.8)	24 (30.8)	13 (27.7)		
Marital status (married, %)	52 (94.5)	68 (87.2)	34 (72.3)	9.582^b^	0.008
≥2 chronic comorbidities (%)	12 (21.8)	30 (38.5)	25 (53.2)	11.375^b^	0.003
Hypertension history (%)	36 (65.5)	63 (80.8)	43 (91.5)	10.519^b^	0.005
NIHSS score [M (P25, P75)]	5.0 (3.0, 8.0)	9.0 (6.0, 14.0)	14.0 (9.0, 18.0)	38.126^c^	<0.001
GCS grade (%)				12.846^b^	0.012
13–15 points	48 (87.3)	62 (79.5)	28 (59.6)		
9–12 points	6 (10.9)	12 (15.4)	14 (29.8)		
3–8 points	1 (1.8)	4 (5.1)	5 (10.6)		
Psychological resilience (CD-RISC, mean ± SD)	68.5 ± 10.2	58.4 ± 9.8	48.2 ± 8.6	51.35^a^	<0.001
Social support (MOS-SSS, mean ± SD)	68.2 ± 8.6	58.7 ± 9.2	49.5 ± 8.8	55.28^a^	<0.001
Fear of disease progression (FoP-Q-SF, mean ± SD)	26.4 ± 6.2	34.8 ± 7.5	42.6 ± 6.8	68.74^a^	<0.001

a Indicates *F*.

b Indicates *χ*^2^ value.

c Indicates *H*-value.

Class 1 = low-level stable fatigue group, Class 2 = moderate-level slowly resolving group, Class 3 = high-level persistent fatigue group.

### 3.4 Multinomial logistic regression analysis of factors influencing post-stroke fatigue developmental trajectories in young and middle-aged patients with intracerebral hemorrhage

Variables with *p* < 0.10 in the univariate analysis (age, educational level, marital status, number of chronic comorbidities, hypertension history, NIHSS score, GCS grade, psychological resilience total score, social support total score, and fear of disease progression total score) were included in a multinomial logistic regression model, with the low-level stable fatigue group (Class 1) as the reference, using the forward stepwise method for variable selection. The Hosmer-Lemeshow test for the multinomial logistic regression model showed good model fit (*χ*^2^ = 8.342, *p* = 0.398).

The results showed that: (1) Compared with Class 1, independent risk factors for entering Class 2 (moderate-level slowly resolving group) included increasing age (OR = 1.085, 95%CI: 1.023–1.151, *p* = 0.007), having ≥2 chronic comorbidities (OR = 2.186, 95%CI: 1.174–4.071, *p* = 0.014), and higher NIHSS score (OR = 1.118, 95%CI: 1.036–1.206, *p* = 0.004); protective factors included higher psychological resilience score (OR = 0.951, 95%CI: 0.920–0.983, *p* = 0.003) and higher social support score (OR = 0.936, 95%CI: 0.895–0.978, p = 0.004). (2) Compared with Class 1, independent risk factors for entering Class 3 (high-level persistent fatigue group) included increasing age (OR = 1.132, 95%CI: 1.054–1.216, *p* = 0.001), having ≥2 chronic comorbidities (OR = 3.418, 95%CI: 1.642–7.114, *p* = 0.001), higher NIHSS score (OR = 1.194, 95%CI: 1.092–1.305, *p* < 0.001), and higher fear of disease progression score (OR = 1.112, 95%CI: 1.046–1.182, *p* = 0.001); protective factors included higher psychological resilience score (OR = 0.924, 95%CI: 0.887–0.963, *p* < 0.001), higher social support score (OR = 0.908, 95%CI: 0.859–0.960, *p* = 0.001), and college degree or above (OR = 0.395, 95%CI: 0.178–0.876, *p* = 0.022) (Table 5).

**Table 5:** Multinomial logistic regression analysis of factors influencing post-stroke fatigue developmental trajectories in young and middle-aged patients with intracerebral hemorrhage.

Variable	B	SE	Wald *χ*^2^	*p*	OR	95% CI
Class 2 vs. Class 1						
Age	0.082	0.030	7.384	0.007	1.085	1.023–1.151
≥2 chronic comorbidities	0.782	0.317	6.076	0.014	2.186	1.174–4.071
NIHSS score	0.112	0.039	8.289	0.004	1.118	1.036–1.206
Psychological resilience (CD-RISC)	−0.050	0.017	8.734	0.003	0.951	0.920–0.983
Social support (MOS-SSS)	−0.066	0.023	8.425	0.004	0.936	0.895–0.978
Class 3 vs. Class 1						
Age	0.124	0.036	11.864	0.001	1.132	1.054–1.216
≥2 chronic comorbidities	1.229	0.374	10.798	0.001	3.418	1.642–7.114
NIHSS score	0.177	0.045	15.537	<0.001	1.194	1.092–1.305
Fear of disease progression (FoP-Q-SF)	0.106	0.031	11.831	0.001	1.112	1.046–1.182
Psychological resilience (CD-RISC)	−0.079	0.021	14.504	<0.001	0.924	0.887–0.963
Social support (MOS-SSS)	−0.096	0.028	11.976	0.001	0.908	0.859–0.960
Educational level (college degree or above)	−0.929	0.406	5.236	0.022	0.395	0.178–0.876

## 4 Discussion

To our knowledge, this is the first longitudinal study employing latent class growth modeling to identify developmental trajectories of post-stroke fatigue specifically among young and middle-aged patients with intracerebral hemorrhage. The findings revealed that fatigue changes did not follow a single pattern but rather exhibited three heterogeneous trajectories: the “low-level stable fatigue group” (30.6%), the “moderate-level slowly resolving group” (43.3%), and the “high-level persistent fatigue group” (26.1%). This finding validates the core tenet of the Chronic Illness Trajectory Framework, namely that disease trajectories exhibit significant individual differences and group heterogeneity (12), and also extends beyond the limitations of previous studies on post-stroke fatigue that were predominantly based on cross-sectional designs or that only reported overall levels of change. These three trajectory classes are consistent with findings from previous studies. Kjeverud et al. (9) identified three latent classes of fatigue trajectories over 18 months post-stroke—low, moderate, and high—using growth mixture modeling. Similarly, Xiong et al. (19) reported three trajectory categories in middle-aged and elderly stroke patients. Huang et al. (8) identified distinct fatigue trajectories in older adults after first ischemic stroke. The consistency of identifying a high-risk persistent fatigue subgroup (approximately 20–30%) across studies underscores the clinical importance of early identification of this vulnerable population.

In this study, more than two-thirds of the patients (69.4%) were classified into Class 2 or Class 3, indicating that the overall burden of post-stroke fatigue is substantial in young and middle-aged patients with intracerebral hemorrhage. Compared with the ischemic stroke population, the mass effect of hematoma and intracranial hypertension during the acute phase of intracerebral hemorrhage may cause more direct damage to the ascending reticular activating system (20), which may explain why patients in Class 3 of this study still had a mean FSS score as high as (5.85 ± 1.04) even at T4 (12 months post-discharge).

It is noteworthy that Class 2 (43.3%) was the largest trajectory class in this study. Patients in this group exhibited a “slowly resolving” pattern, with FSS scores gradually decreasing from (5.62 ± 0.88) at T1 to (3.75 ± 1.08) at T4, indicating that fatigue in most young and middle-aged patients with intracerebral hemorrhage gradually improves with prolonged rehabilitation time, but this process is relatively lengthy and may persist up to 12 months post-discharge. This finding is consistent with Juárez-Belaúnde et al.’s (5) observation that fatigue tends to ameliorate over time in hemorrhagic stroke cases, paralleling functional recovery. Class 1 (30.6%) maintained consistently low fatigue levels throughout, which may be attributed to patients’ favorable baseline neurological status (median NIHSS score of 5.0) and higher psychosocial resource reserves. The three trajectories identified in this study share similarities with previous findings. The identification of a “high-level persistent fatigue” high-risk subtype (approximately 20–30%) is consistent across studies; the difference is that the “moderate-level slowly resolving group” accounted for a higher proportion (43.3%) in this study, which may be related to the inclusion of patients with intracerebral hemorrhage and the 12-month follow-up period (8, 14). The neurological recovery window for intracerebral hemorrhage is longer than that for ischemic stroke (21, 22), and the alleviation of fatigue consequently exhibits a more gradual downward slope. This finding suggests that clinical nursing staff should maintain reasonable expectations regarding fatigue recovery in young and middle-aged patients with intracerebral hemorrhage, avoiding unnecessary anxiety or premature termination of interventions due to a lack of significant improvement in the short term.

On the basis of identifying the three heterogeneous trajectories, this study further analyzed the factors influencing patients’ allocation to different trajectory classes. Regarding demographic factors, increasing age was an independent risk factor for entering both Class 2 (OR = 1.085, *p* = 0.007) and Class 3 (OR = 1.132, *p* = 0.001), a finding consistent with previous studies (23). With increasing age, the decline in neural plasticity and elevated levels of inflammatory responses and oxidative stress may lead to more severe fatigue perception (24). Notably, the age range in this study was only 22 to 59 years, yet the effect of age remained significant even within this relatively narrow “young and middle-aged” range, suggesting that clinicians should pay particular attention to fatigue management in older young and middle-aged patients with intracerebral hemorrhage (e.g., those aged 50–59 years). Regarding educational level, having a college degree or above was a protective factor against entering Class 3 (OR = 0.395, *p* = 0.022), but had no significant effect on entering Class 2. Patients with higher education may possess stronger disease awareness and information-seeking abilities, enabling them to participate more actively in rehabilitation decisions and effectively manage their own symptoms (25). At the same time, higher educational levels are often associated with more flexible psychological coping strategies, which may help buffer the impact of disease on self-efficacy (26). This finding suggests that nursing staff should adopt more intuitive and accessible health education approaches for young and middle-aged patients with low educational levels, and appropriately increase follow-up frequency. Marital status showed a statistically significant difference in univariate analysis (*p* = 0.008), with married patients accounting for a higher proportion across all groups, which may be related to the overall high marriage rate (85.6%) in this study population. However, marital status did not enter the final multivariate model, suggesting that its effect may be mediated by other variables such as social support, which is consistent with the theoretical framework of social support (27).

In terms of disease severity, elevated NIHSS score was an independent risk factor for entering Class 2 (OR = 1.118) and Class 3 (OR = 1.194), with a stronger effect on Class 3. Greater severity of neurological deficits implies more extensive brain tissue damage and a more prolonged rehabilitation process, during which patients need to expend greater physical and mental effort in the recovery of motor function (5), directly contributing to the exacerbation and persistence of fatigue. This aligns with Scopelliti et al.’s finding that more severe neurological deficits are associated with higher fatigue burden in ICH survivors (11). Concurrently, having two or more chronic comorbidities had significant effects on both Class 2 (OR = 2.186) and Class 3 (OR = 3.418), with a substantially higher OR in Class 3, demonstrating a dose–response relationship. Hypertension (78.9%) and diabetes (28.3%) were the most prevalent comorbidities in this study. These chronic conditions can themselves induce fatigue through mechanisms such as chronic inflammation and mitochondrial dysfunction (28). Consistent with these findings, Kjeverud et al. reported that having multiple comorbidities was significantly associated with higher fatigue trajectories in both univariate and multivariable analyses. For young and middle-aged patients with intracerebral hemorrhage and multiple comorbidities, nursing staff should implement a multimorbidity management model, integrating blood pressure and blood glucose control with fatigue interventions. GCS grade showed in univariate analysis that the proportion of patients with moderate-to-severe consciousness impairment (GCS < 9) in Class 3 (10.6%) was higher than that in Class 1 (1.8%), but it did not enter the final multivariate model. This may be due to collinearity between GCS and NIHSS (both reflecting disease severity), or it may suggest that the effect of consciousness impairment on fatigue is primarily mediated through neurological deficits.

Regarding psychosocial factors, higher psychological resilience score was a protective factor for both Class 2 (OR = 0.951) and Class 3 (OR = 0.924), with a stronger protective effect on Class 3. Psychological resilience reflects an individual’s capacity for adaptation and recovery in the face of trauma and adversity (29). Patients with high psychological resilience tend to adopt positive coping strategies such as problem-solving and seeking informational support, rather than passive avoidance or catastrophic thinking (30), which may reduce the subjective perception of fatigue at the cognitive level. This finding aligns with previous research demonstrating that resilience is independently associated with better psychological outcomes in stroke survivors (13). Higher social support score similarly had protective effects on both Class 2 (OR = 0.936) and Class 3 (OR = 0.908), with a lower OR in Class 3, suggesting that the protective effect of social support was more prominent in the high-level persistent fatigue group. Corresponding to the above protective factors, higher fear of disease progression score was an independent risk factor for entering Class 3 (OR = 1.112), but had no significant effect on Class 2. Fear of disease progression refers to an individual’s excessive worry and fear about disease recurrence, progression, and sequelae (31). Young and middle-aged patients with intracerebral hemorrhage face realistic pressures such as returning to work and assuming the role of family economic provider, and fear of disease recurrence and sequelae may continuously activate the hypothalamic–pituitary–adrenal axis, leading to energy depletion under chronic stress (32, 33), ultimately manifesting as persistent severe fatigue. This finding suggests that clinical nursing interventions should not focus solely on the symptom of fatigue itself, but should also screen for and address patients’ fear of disease progression. Cognitive behavioral therapy and mindfulness-based interventions may help break the vicious cycle of “fear-stress-fatigue.” It is noteworthy that psychological resilience, social support, and fear of disease progression are closely interrelated: higher social support and psychological resilience can buffer the psychological impact of illness and reduce fear levels (34), while reduced fear in turn facilitates energy recovery. This suggests that in clinical practice, interventions for fatigue in young and middle-aged patients with intracerebral hemorrhage should construct a comprehensive psychosocial support model of “enhancing psychological resilience, strengthening social support, and alleviating fear of disease progression,” rather than targeting any single factor in isolation. It is important to acknowledge that the psychosocial factors (psychological resilience, social support, and fear of disease progression) were measured only at baseline. While this study demonstrates associations between these factors and fatigue trajectory membership, causal relationships cannot be inferred. The observed associations may reflect bidirectional relationships, where individuals with less severe fatigue are better able to maintain social connections and psychological well-being, or they may be influenced by unmeasured confounding variables. Future studies employing repeated measurements of these psychosocial factors are needed to examine their dynamic interplay with fatigue over time.

The above findings have clear implications for clinical nursing practice in young and middle-aged patients with intracerebral hemorrhage. First, fatigue assessment should be dynamic and individualized. Given the significant individual differences in fatigue trajectories, nursing staff should conduct standardized fatigue assessments (e.g., using the FSS) at key time points including the acute phase, 1 month, 6 months, and 12 months post-discharge, rather than relying solely on a single assessment at admission. This approach aligns with the consensus-based core recommendations from the third Stroke Recovery and Rehabilitation Roundtable (SRRR), which advocate that everyone who has had a stroke should be assessed for fatigue and that the Fatigue Severity Scale be used as an outcome measure in research. For screened high-risk populations (those with high NIHSS scores, multiple comorbidities, and low psychological resilience), more intensive follow-up plans should be developed. Second, intervention strategies should be stratified and precise. For patients in Class 3 (high-level persistent fatigue), psychosocial interventions (e.g., cognitive behavioral therapy and mindfulness-based stress reduction) and fear of disease progression management should be prioritized, along with multidisciplinary team coordination (neurology, rehabilitation, and psychology) to provide comprehensive support. However, it should be noted that while psychosocial factors such as fear, stress, and anxiety were partially assessed in this study (via the FoP-Q-SF), comprehensive assessment of depression and other mood disorders was not performed. Therefore, recommendations regarding psychosocial interventions should be considered hypothesis-generating and grounded in existing literature rather than derived directly from the present data. For patients in Class 2 (moderate-level slowly resolving), interventions may focus on energy conservation strategy education and progressive rehabilitation training to help patients allocate energy appropriately during the recovery process. In addition, the special needs of young and middle-aged patients should be addressed. These patients have more urgent needs for return to work and social role reintegration. Nursing staff should incorporate work ability assessment and vocational rehabilitation counseling into routine care processes, helping patients set realistic recovery goals and avoiding frustration and exacerbated fatigue caused by overly high expectations.

This study has several limitations. First, this study only included a single-center sample, and although the sample size (180 cases) met the requirements for LCGM model fitting, it was relatively small and may have limited the ability to identify rare trajectory subtypes. The generalizability of the findings requires further validation through multicenter, large-sample studies. Second, the follow-up period of this study was 12 months, which, while covering the main window of neurological recovery after intracerebral hemorrhage, cannot answer whether fatigue would further resolve or exhibit new changing trends over a longer period (e.g., 2–3 years). rehabilitation training intensity, and depression, which is a well-established predictor of post-stroke fatigue, that may play important roles in the occurrence and maintenance of post-stroke fatigue. Notably, Scopelliti et al. (11) found that depressive symptoms were the only clinical factor significantly associated with 1-year fatigue in ICH survivors in multivariable analysis, underscoring the importance of depression assessment in future research. The exclusion of depression as a predictor is a notable limitation, as previous studies have consistently demonstrated its association with fatigue. Fourth, this study used scales to assess fatigue, which are subjective report measures and lack objective physiological indicators (e.g., inflammatory cytokine levels and neurophysiological parameters) for corroboration. Future studies may consider combining subjective and objective measures. Fifth, the psychosocial factors (psychological resilience, social support, and fear of disease progression) were assessed only at baseline. This cross-sectional measurement of predictors limits the ability to examine how changes in these factors over time may influence fatigue trajectories. Future research should incorporate repeated measurements of these psychosocial variables to capture their dynamic relationships with fatigue. Sixth, there were some differences in sample sizes across trajectory groups (55 cases in Class 1, 78 cases in Class 2, and 47 cases in Class 3). Although LCGM can handle unbalanced data, this may affect statistical power in subsequent regression analyses.

## 5 Conclusion

There are three heterogeneous developmental trajectories of post-stroke fatigue in young and middle-aged patients with intracerebral hemorrhage: the low-level stable fatigue group (30.6%), the moderate-level slowly resolving group (43.3%), and the high-level persistent fatigue group (26.1%). Increasing age, a higher number of chronic comorbidities, more severe neurological deficits, and higher levels of fear of disease progression are independent risk factors for entering high-level fatigue trajectories, while psychological resilience, social support, and higher educational level are important protective factors. Clinical nursing should implement stratified, dynamic, and precise interventions based on different trajectory characteristics, with particular attention to the psychosocial needs of high-risk populations. Clinicians should routinely assess psychological resilience and social support during rehabilitation planning, as these factors may serve as important targets for future intervention research to promote effective management of post-stroke fatigue and comprehensive recovery of social function.

## Data Availability

The raw data supporting the conclusions of this article will be made available by the authors, without undue reservation.
